# Glial Activation, Neuroinflammation, and Loss of Neuroprotection in Chronic Pain: Cellular Mechanisms and Emerging Therapeutic Strategies

**DOI:** 10.3390/biomedicines14010058

**Published:** 2025-12-26

**Authors:** Alyssa McKenzie, Rachel Dombrower, Nitchanan Theeraphapphong, Sophia McKenzie, Munther A. Hijazin

**Affiliations:** 1 School of Medicine, St. Georges University, University Centre Grenada, West Indies 11739, Grenada; 2 School of Medicine, Uniformed Services University of the Health Sciences, Bethesda, MD 20814, USA; 3 Prentice, Mitri & Hijazin Neurological Associates, Downey, CA 90241, USA; 4 Keck School of Medicine, University of Southern California, Los Angeles, CA 90033, USA

**Keywords:** microglia, astrocytes, satellite glial cells, glial activation, neuroinflammation, neuroprotection, neuroimmune signaling, reactive gliosis, glial-neuron interactions, chronic pain

## Abstract

Chronic pain is increasingly regarded as a condition of glia–neuronal dysregulation driven by persistent neuroinflammatory signaling. Following injury to nerves or tissues, glial cells, including astrocytes or satellite glial cells, undergo changes in their phenotype, thereby amplifying painful stimuli mediated by cytokines, chemokines, or ATP signaling. In response to injuries, activated microglia release several mediators such as BDNF, IL-1β, or TNF-α, thereby disrupting chloride homeostasis and inducing disinhibition in the dorsal horn, and sustaining maladaptive neuroimmune activity. Dysfunction of astrocytes, characterized by impaired glutamate clearance via excitatory amino acid transporter 2 and elevated C-X-C motif chemokine ligand 1 (CXCL1) and ATP release, drives neuronal sensitization, loss of neuroprotective metabolic support, and persistence of pain. In peripheral ganglia, connexin–43–mediated satellite glial cell coupling leads to hyperexcitability, resulting in neuropathic and orofacial pain and contributing to peripheral neuroinflammation. Presently, there is no unified framework for glial cell types, and the molecular mechanisms underlying microglial, astrocyte, and satellite glial cell contributions to the transition to chronic pain from acute pain are not completely elucidated. This review synthesizes current evidence on cellular and molecular mechanisms linking glial reactivity to pain chronification through sustained neuroinflammatory remodeling and impaired neuroprotection. It evaluates therapeutic strategies, including purinergic receptor P2X4 and toll-like receptor 4 antagonists, to metabolic reprogramming, exosome therapy, and neuromodulation, aimed at restoring homeostatic glial function and re-establishing neuroprotective glia–neuron interactions. A deeper understanding of the temporal and spatial dynamics of glial activation may enable personalized, non-opioid interventions that not only achieve durable analgesia but also prevent progressive neuroinflammatory damage and support long-term functional recovery.

## 1. Introduction

Chronic pain is currently considered a maladaptive neuroimmune state perpetuated by complex glia-mediated mechanisms affecting both the peripheral nervous system (PNS) and central nervous system (CNS) [1,2,3,4]. Under physiologic conditions, glial cells, such as astrocytes, microglia, and satellite glia, maintain homeostasis and regulate synaptic and neuronal function through essential neuroprotective, metabolic, and anti-inflammatory support [3,5,6,7]. However, injury or neuronal stress can induce glial activation, producing phenotypes that amplify nociceptive signaling while simultaneously diminishing these neuroprotective functions [8]. When this activation becomes persistent, these cells sustain a pathological neuroinflammatory state characterized by impaired neuronal protection, disrupted metabolic coupling, and chronic cytokine dysregulation, central to chronic pain maintenance [5,6,9,10].

The public health implications are considerable, including that chronic pain affects approximately 20% of populations globally, remaining a leading cause of disability [11,12]. Glial-driven neuroimmune amplification also contributes to reduced opioid efficacy by interfering with opioid receptor signaling and promoting tolerance and neuroinflammation [2,4,13]. Foundational work, including that by Grace et al. [4], highlights dysregulated glia–neuron interactions as convergent mechanisms across chronic pain conditions [2].

However, despite considerable advances, current knowledge remains fragmented across microglia, astrocytes, and satellite glial cells, and the field still lacks an integrated framework explaining how glial activation across the PNS and CNS collectively drives the transition from acute pain to chronic pain through sustained neuroinflammatory remodeling and progressive loss of neuroprotective homeostasis [3,4,5,14].

Accordingly, this review discusses how acute glial cell responses to injury evolve into their dysregulated states in chronic disease, specifically focusing on microglia, astrocytes, and satellite glial cells [3,4,5,14]. Some key mediators of these maladaptive neuroimmune interactions include toll-like receptor 4 (TLR4), P2X4 purinergic receptor, mitogen-activated protein kinases (MAPKs), and brain-derived neurotrophic factor (BDNF) [3,15,16,17]. Finally, we discuss emerging therapeutic strategies targeting glial modulation and neuroimmune interactions to treat chronic pain with particular attention to restoring neuroprotective glial functions and mitigating chronic neuroinflammation [13,18].

To support the scope of this review, a targeted literature search of PubMed, Scopus, and Web of Science was conducted using combinations of keywords including microglia, astrocytes, satellite glial cells, neuroinflammation, central sensitization, and chronic pain. Peer-reviewed articles addressing mechanistic, translational, and therapeutic aspects of glial interactions in chronic pain were prioritized, and reference lists of key publications were reviewed to identify additional relevant studies.

## 2. Microglia: Initiators of Central Sensitization

### 2.1. Activation Triggers

**Figure 1:**
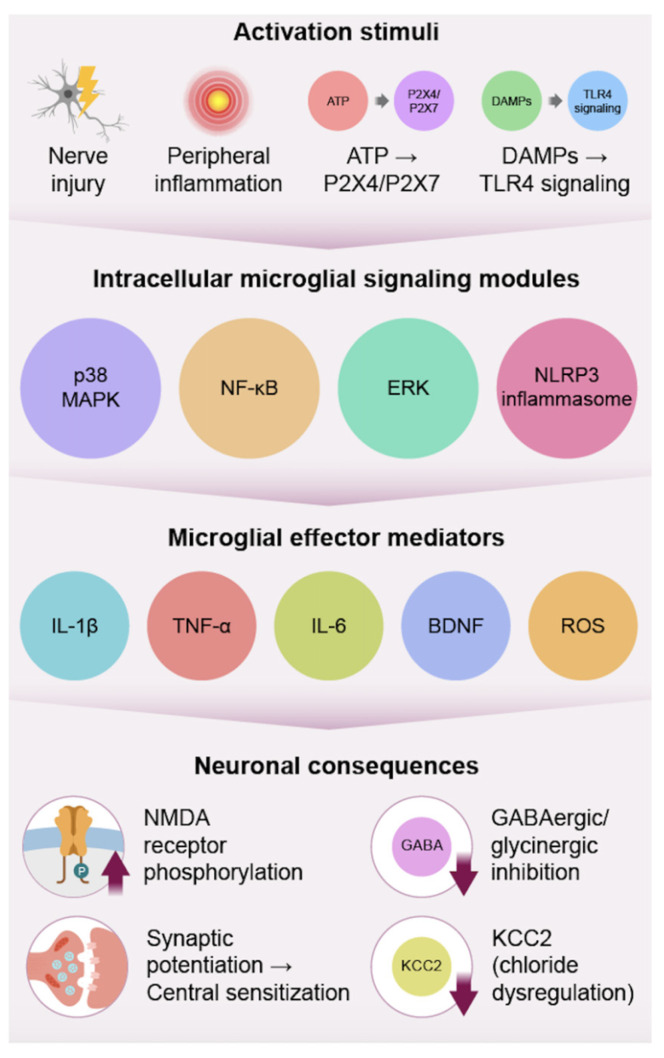
Microglial activation pathway. Microglia respond to nerve injury and peripheral inflammation through activation of receptors such as P2X4/P2X7 and TLR4, which detect ATP and DAMPs. These signals trigger intracellular pathways including p38 MAPK, NF-κB, ERK, and the NLRP3 inflammasome, leading to the release of inflammatory mediators (IL-1β, TNF-α, IL-6), BDNF, and ROS. These effectors drive neuronal hyperexcitability through NMDA receptor phosphorylation, reduced GABAergic inhibition, and KCC2 dysregulation, promoting central sensitization and chronic pain.

Microglia are the primary innate immune sentinels in the CNS, and are among the earliest glial cell types to respond to injury to peripheral nerves. Tissue injury initiates the release of various danger signals, such as ATP, HMGB1, and other danger-associated molecular patterns, which activate microglia through interacting with their characteristic pattern recognition receptors, including TLR4, and the P2X4 and P2X7 receptor subunits, thereby initiating a rapid microglial activation [3,16]. Neuronal CX3CL1 also plays a critical role in interacting with its receptor, CX3CR1, on microglia, thereby facilitating chemotaxis, proliferation, and enhanced sensitivity to other stimuli [3]. Microglia in the dorsal horn of the spinal cord undergo pronounced morphological changes, ranging from acting as surveillance cells to phagocytic cell types, and begin proliferating within hours of injury, with cell expansion usually peaking within 1–3 days [19]. These early responses to injury, which establish a pro-inflammatory environment preceding astrocyte response, contribute directly to the development of central sensitization [3]. Although typically involved in debris clearance and metabolic support under physiological conditions, persistent activation shifts microglia from homeostatic, neuroprotective phenotypes to maladaptive pro-inflammatory states that drive sustained neuroinflammation [17,19,20]. The stimuli trigger microglia to initiate the earliest neuroimmune responses that drive the transition from acute to chronic pain, illustrated in Figure 1 [1,3].

### 2.2. Effector Pathways

Following activation, microglia communicate with downstream intracellular signaling pathways, which facilitate the establishment and maintenance of central sensitization [1]. Stimulation via p38-MAPK and NF-κB activates gene transcription to produce IL-1β, TNF-α, and IL-6, which facilitate increased excitatory neurotransmission and decreased inhibitory neurotransmission in the dorsal horn neurons [1,16]. Microglia also release BDNF, which binds to TrkB receptors on neurons to reduce the activity of the chloride transport protein, KCC2. This results in an overall depolarization shift in GABA neurotransmission, ultimately weakening the inhibitory functions [15]. Other molecules produced by microglia, such as ATP and reactive oxygen species (ROS), further enhance synaptic excitability and nociceptive functionality [1,16]. Sustained microglial activation also disrupts neuroprotective homeostasis by promoting mitochondrial dysfunction, oxidative stress, and impaired phagocytic clearance, further amplifying neuroinflammatory signaling [17,20,21]. Notably, microglial signaling is sexually dimorphic, with TLR4-dependent mechanisms primarily dominant in males, whereas T-cell-mediated mechanisms compensate in females, underscoring the need for sex-specific therapeutic interventions to facilitate efficient treatment [22]. By activating these effector pathways, microglia help establish an efficient pro-inflammatory environment that reinforces neuronal hyperexcitability. Human neuroimaging studies support these findings, with TSPO-PET demonstrating elevated microglial activation in chronic pain patients [23].

### 2.3. Resolution and Modulation

Though microglia are implicated in the maintenance of chronic pain, they have also been shown to have the capacity to switch to pro-resolving phenotypes in optimized settings [19]. Stimulation of anti-inflammatory cytokines, including IL-4 and IL-10, combined with PPAR-γ stimulation, facilitates microglia switching from an M1 to an M2 phenotype, supporting tissue repair, neuroprotection, and restoration of synaptic homeostasis [19]. M2 microglia produce growth factors and anti-inflammatory mediators to restore normal neurotransmission in addition to preventing dorsal horn hypersensitivity [1,19]. The ability of microglia to transition into neuroprotective phenotypes highlights their dual role in CNS injury, where failure to resolve activation contributes to chronic neuroinflammation and long-term neuronal vulnerability [19]. The usage of pharmacological interventions targeting microglial activation has proven to be therapeutic in clinical settings [24]. Thus, agents like minocycline reduce microgliosis and cytokine response, while CSF1R inhibitors effectively suppress microglia proliferation [25,26,27,28]. Such interventions not only inhibit neuroinflammation, alleviating neuropathic pains, but also inhibit pain in preclinical models [25,26]. Therapeutic manipulation in microglial phenotypes could, therefore, be a strategy for preventing the transition of acute to chronic pain.

### 2.4. Oligodendrocyte Precursor Cells and Neuroinflammatory Signaling

Oligodendrocyte precursor cells (OPCs) have additionally been recognized as active participants in neuroinflammatory signaling rather than passive progenitors of myelinating oligodendrocytes [29]. In instances of CNS injury, activated microglia release cytokines and growth factors that influence OPC proliferation, differentiation, and survival [29,30]. These OPCs express inflammatory receptors that respond directly to cytokines such as IL-1β and TNF-α, which may impair normal myelination and contribute to altered axonal conduction and network excitability [30,31]. Emerging evidence suggests that sustained neuroinflammation can arrest OPC maturation, resulting in dysfunction of glial interaction that perpetuates central sensitization [29]. Although the role of OPC in chronic pain has yet to be fully defined, OPC dysfunction represents an additional mechanism through which neuroinflammation contributes to the destabilization of nervous system circuitry in chronic pain conditions.

## 3. Astrocytes: Sustainers of Pain Chronification

### 3.1. Reactive Transformation

After repeated nociceptive stimulation, the transition of astrocytes to reactive phenotypes plays a central role in maintaining chronic pain. These reactive states are characterized by their enhanced immunoreactivity for GFAP, vimentin, and S100β, indicating cytoskeletal reorganization and increased energy demand [6,9,18]. Simultaneously, astrocytes also downregulate excitatory amino acid transporter 2 and inward-rectifying potassium channel 4.1, impairing glutamate uptake and potassium buffering [5,6]. These alterations lead to extracellular accumulation of glutamate and potassium, which enhances excitatory neuronal firing [5,18]. Reactive astrocytes additionally increase their release of ATP, D-serine, and CXCL1, which are gliotransmitters, and these further facilitate N-methyl-D-aspartate receptor signaling and promote long-lasting synaptic plasticity associated with central sensitization [5,18]. Beyond these excitatory effects, prolonged astrocytic reactivity reflects a shift from their normal neuroprotective metabolic support roles to maladaptive inflammatory phenotypes that destabilize neuronal homeostasis. Collectively, these modifications heighten dorsal horn excitability, supporting the transition from acute to chronic pain. Thus, reactivity of astrocytes represents an essential linkage between early microglial activation and sustained neuroimmune dysregulation. Astrocytic activation within the lumbar dorsal horn has also been implicated in chronic low back pain through sustained glutamatergic dysregulation and persistent central sensitization [5,18].

### 3.2. Intracellular Signaling

Reactive astrocytes sustain chronic pain by activating numerous intracellular pathways to maintain their pro-inflammatory status [9,18]. STAT3 and JAK stimulation maintain hypertrophic, metabolic, and cytokine-secretory programs through transcription, creating a chronic reactive state [17,18,20]. NF-κB activation also leads to IL-1β, TNF-α, and chemokine production, which in turn contribute to both microglial infiltration and neuronal sensitization [17,20]. Another critical pathway involves the activation of NLRP3 inflammasomes, which enhances IL-1β maturation and amplifies local inflammatory signaling [21,24,25]. Concomitantly, mitochondrial dysfunction and ROS increase astrocyte reactivity by promoting oxidative injury, thereby disrupting energy regulation [17,20]. Sustained activation of these intracellular pathways not only drives neuroinflammation but also undermines astrocytes’ neuroprotective capacities, limiting metabolic coupling and impairing antioxidant defense mechanisms [18]. Intracellular interactions in these reactive astrocytes create an autogenous, self-sustaining, feed-forward loop, maintaining astrocytes in a chronically active state even after the initial injury has resolved [17,18].

### 3.3. Cross-Talk with Microglia & Neurons

**Figure 2:**
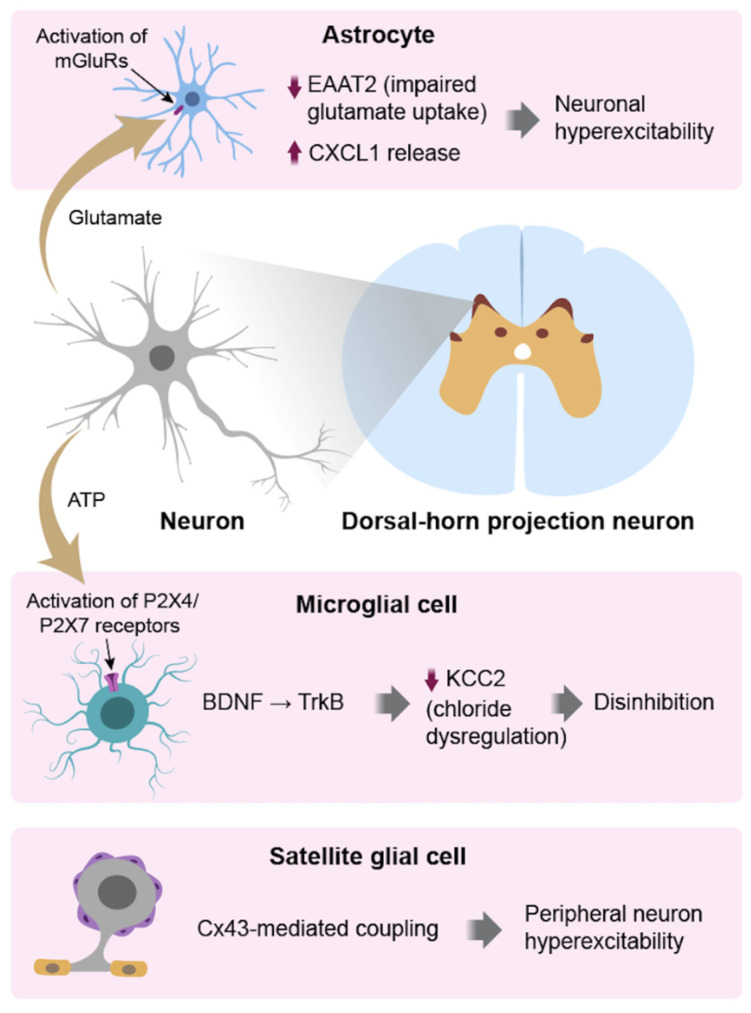
Glia-neuron cross-talk. Astrocytes, microglia, and satellite glial cells interact bidirectionally with neurons to promote hyperexcitability. Astrocytic EAAT2 downregulation and CXCL1 release increase excitatory signaling. Microglial activation through P2X4/P2X7 receptors induces BDNF–TrkB signaling and KCC2 downregulation, causing disinhibition. Satellite glial cell Cx43-mediated coupling enhances peripheral neuron excitability. These interactions create a feed-forward loop contributing to chronic pain.

Astrocytes contribute to chronic pain via extensive bidirectional communication with microglia and neurons. IL-6 and MCP-1 derived from astrocytes maintain microglia activation, thereby establishing persistent pro-inflammatory interactions that sensitize the CNS [5,18]. Conversely, microglia-derived cytokines induce astrocyte activation, creating a synergistic glial feedback loop that sustains neuroimmune dysregulation [5,18]. At the neuronal levels, tripartite synapse dysfunction, in which astrocytes regulate neurotransmitter reuptake, results in glutamatergic hyperactivity and decreased inhibition [5,18]. Astrocytes also modulate synaptogenesis and spine remodeling, contributing to the maintenance of structural changes involved in chronic pain circuits [5,13]. Through these interactions, reactive astrocytes lose their normal neuromodulatory and neuroprotective roles and instead perpetuate chronic neuroinflammation and circuit-level instability [5,18]. These interactions establish astrocytes as key mediators of molecular, cellular, and synaptic alterations in chronic pain, illustrated in Figure 2 [5,18].

## 4. Satellite Glial Cells (SGCs) in the Periphery

SGCs form a densely interconnected sheath around primary sensory neurons in the dorsal root ganglia (DRG) and trigeminal ganglia, functioning as an essential role in regulating excitability and sensitization in primary sensory neurons [7,32]. Initially, these cells were considered to be passive support cells; however, SGCs are now known to have an active role in pain signaling that relies on rapid communication with sensory fibers via gap junctions, specifically connexin-43 (Cx43), with particular emphasis on cytokine and purinergic signaling pathways [28,32,33]. Upon tissue injury or inflammation, these glial cells become reactive, with characteristic features including increased glial markers, enhanced gap junction coupling, and enhanced release of inflammatory mediators, all having a direct effect on sensory neuron membrane properties [28,32]. These adaptations are primarily responsible for contributing to the pathologic properties observed in primary sensory neurons of the peripheral ganglia [28,32]. Consequently, glial cell activation has now been identified as an underlying major initiator in persistent types of pains, such as neuropathic, inflammatory, and orofacial pains [28,32]. Importantly, persistent SGC reactivity contributes to peripheral neuroinflammation and loss of normal neuroprotective buffering functions within the ganglia, increasing neuronal vulnerability [28,32].

Beyond spinal nociception, SGC activation within the trigeminal ganglion has also emerged as a contributor to headache pathogenesis, in which neuroimmune signaling and gap-junction coupling amplify trigeminovascular excitability and maintain pain chronification. The interaction between these glial and neuronal cells provides a bridge between local inflammation and sustained central sensitization relevant to migraine and other chronic headache disorders [34].

### 4.1. Reactive Changes and Neuroimmune Signaling

Following injury to the peripheral nerve, SGCs produce IL-1β, IL-6, TNF-α, and ATP, and each of these potentiate sensory neuronal depolarization and the likelihood of action potential firing [14,32,33]. ATP stimulation of P2X and P2Y receptors on sensory neurons promotes excitatory activity, while IL-6, IL-1β, and TNF-α stimulate changes in ion channel expression, further increasing neural responsiveness [28,32]. Reactive SGCs also upregulate Cx43, which increases coupling between SGCs and facilitates neural–glial communication over extensive areas of the sensory ganglia [28]. These mechanisms create a multicellular amplification circuit through which inflammatory signals are transmitted from sensory neurons to glia, significantly heightening the region of peripheral sensitization from injury to the distant region of the nerve [28,33]. This neuroimmune amplification not only sustains peripheral neuroinflammation but also imposes metabolic stress on sensory neurons by disrupting potassium and glutamate homeostasis, thereby weakening local neuroprotective control mechanisms [28,33]. These mechanisms establish the crucial role of SGCs in maintaining neural inflammation in the periphery.

### 4.2. Macrophage–SGC Cross-Talk

Macrophage entry into the DRG is a characteristic component of neuropathic and inflammatory pain, which interacts closely with SGC activation [32,33]. IL-1β, TNF-α, and CCL2 released from macrophages increase SGC reactivity, whereas activated SGCs produce chemokines to attract more macrophages, creating an inflammatory bidirectional loop [28,32]. These interactions increase neuronal excitability, resulting in ectopic firing in the DRG, which underlies the mechanism of spontaneous pain. In models of orofacial pain, macrophage–SGC interactions in the trigeminal ganglion have been demonstrated to significantly exacerbate mechanical hypersensitivity [32]. This sustained macrophage–SGC loop further erodes neuroprotective homeostasis within the ganglion and can propagate retrograde neuroinflammatory signaling toward the spinal cord, reinforcing central sensitization [32]. Disrupting this signaling axis in preclinical models decreases both inflammation and pain, highlighting their therapeutic value [32].

### 4.3. Functional Impact on Sensory Neurons and Pain States

SGCs are implicated in regulating numerous functions in neural cells, such as glutamate homeostasis, potassium buffering, and regulation of ion channel expression [28]. Upon activation, these functions are reversed, resulting in potassium ion buildup in the extracellular environment, failure to remove neurotransmitters, and dysfunctional neural activity [28]. Cross-excitation among neural cells via coupled SGC activity leads to neural cell sensitization within the ganglion [28,32]. In chronic disorders, persistent SGC activation leads to increased nociceptive input to the spinal cord, reinforcing the mechanism of central sensitization for chronic pain [7,32]. Loss of neuroprotective buffering and chronic metabolically stressful conditions within the DRG further destabilize neuronal firing patterns and enhance ascending neuroinflammatory signaling [28,32]. These findings establish the critical roles of SGCs in regulating mechanisms in creating neural-immune functions in chronic pain conditions, as demonstrated in Table 1.

## 5. Integrated Glial Crosstalk in Pain Circuits

Chronic pain emerges from an integrated, multicellular neuroimmune network in which microglia, astrocytes, and satellite glial cells (SGCs) dynamically reinforce one another to couple peripheral sensitization with central sensitization into a single pathological circuit [3,4,7]. Following nerve injury, microglia rapidly release IL-1β, TNF-α, and complement factors that induce A1 neurotoxic astrocytes, suppressing glutamate uptake and potassium buffering while promoting synaptic hyperexcitability [35].

These reactive astrocytes subsequently sustain microglial activation by releasing MCP-1/CCL2 and IL-6, creating a persistent central inflammatory loop that amplifies dorsal horn excitability [36,37]. In parallel, peripheral injury activates SGCs in the dorsal root ganglia, leading to upregulation of connexin-43 and strengthened gap-junction coupling, which enhances ATP release and promotes sensory neuron hyperexcitability [38,39]. Macrophages recruited to the DRG intensify this peripheral loop through IL-1β, TNF-α, and CCL2, further increasing SGC reactivity in a manner analogous to microglia–astrocyte interactions in the spinal cord [40,41].

These PNS signals converge centrally, where microglial BDNF-mediated downregulation of KCC2 converts GABAergic signaling from inhibitory to excitatory [42], while astrocytic loss of EAAT2 enables glutamate spillover and prolonged NMDA receptor activation [43]. Concurrently, SGC-mediated disturbances in potassium and glutamate buffering elevate the firing probability of DRG neurons, thereby sustaining high-frequency nociceptive input into ascending spinal pathways [32,44]. In both the PNS and CNS, these cross-talk events not only exaggerate the inflammatory responses but also represent a progressive loss of physiological glial homeostatic functions, further destabilizing neuronal and synaptic circuits and contributing to pain chronification [4,5,7].

Together, these glial–glial and glial–neuronal communication loops establish a unified, self-propagating neuroinflammatory circuit in which peripheral sensitization continuously reinforces central sensitization, defining chronic pain as a systems-level disorder driven by coordinated PNS–CNS glial network dysfunction [4,32,44]. Recognizing chronic pain as a product of coordinated glial network dysfunction highlights the need for interventions that modulate glial signaling and restore neuroimmune balance.

## 6. Therapeutic Strategies

The increased understanding that sustained activation of glial cells drives chronic pain has incentivized the development of novel, mechanism-based therapeutic approaches. Traditional opioid analgesics, while capable of providing short-term relief, are limited by tolerance, dependence, respiratory depression, and hyperalgesia, mediated by the activation of glia via TLR-4 [45]. In contrast, glial-targeted therapies aim to inhibit these neuroinflammatory pathways, thus providing a more durable and disease-modifying pain relief [46,47]. Emerging therapies focus on targeting microglial cells, astrocytes, and SGCs in an effort to restore disrupted neuroprotective homeostasis involved in peripheral and central sensitization [32,47], as summarized in Table 2.

### 6.1. Microglial-Targeted Therapies

Following nerve injury, the major contributors to initiation of neuroinflammation are microglia, which rapidly release IL-1β, TNF-α, BDNF, and complement factors that promote central sensitization [15,16]. Several microglia-directed therapies have shown promise, such as minocycline, CSF1R inhibitors, and inflammasome inhibitors [22,48]. Minocycline, a derivative of the antibiotic tetracycline, has been shown to inhibit microglial activation, cytokine release, and neuropathic pain behaviors in animal models [48,49]. In addition to its anti-inflammatory actions, tetracycline derivatives such as minocycline have been reported to modulate neurotrophic signaling, including effects on BDNF-related pathways, which may contribute to context-dependent effects on neuronal plasticity and pain processing [50]. Some studies also suggest peripheral effects via sodium channel inhibition in dorsal root ganglion neurons [51]. Although results in human studies are less consistent, there appear to be benefits in specific neuropathic conditions [24,52]. CSF1R inhibitors inhibit microglial proliferation provoked by injury and lead to reduced microgliosis and analgesia in various models of pain [27]. Notably, the FDA-approved CSF1R inhibitor pexidartinib, originally developed for tenosynovial giant cell tumor, has recently demonstrated analgesic activity through its ability to dampen microglial proliferation and neuroinflammatory signaling [53]. Inhibitors of the inflammasome targeting NLRP3 can reduce IL-1β processing and the subsequent inflammatory cascade, providing another possible approach to mitigating microglia-mediated sensitization [17,20,54,55].

Beyond these agents, a broader class of microglial targets has also shown preclinical promise, supporting the concept that modulating glial activation can meaningfully alter pain states. There has been sufficient preclinical evidence supporting the value of microglial targets in modulating pain, including TLR4 inhibitors that inhibit DAMP-triggered microglial activation [56,57], while antagonists of the purinergic receptors P2X4 and P2X7 blunt ATP-driven microglial excitability [58,59]. Additionally, NF-κB inhibitors contribute to repression of the overlapping inflammatory transcriptional mechanisms of microglia and astrocytes [60,61,62].

### 6.2. Glucocorticoids and Glial Modulation in Chronic Pain

Glucocorticoids, including dexamethasone, prednisone, and methylprednisolone, are widely prescribed anti-inflammatory and immunosuppressive agents used in select chronic pain conditions [63]. Within the central nervous system, they modulate glial activity by suppressing pro-inflammatory cytokine production, inhibiting microglial activation, and altering astrocytic signaling pathways [64,65]. Although their long-term use in chronic pain is constrained by systemic adverse effects and limited disease specificity, their central glial effects highlight the importance of neuroimmune modulation and encourage the development of more selective, glial-directed therapeutic strategies.

### 6.3. Astrocytic-Targeted Therapies

Astrocytes participate in the maintenance of chronic pain due to the dysregulation of glutamate uptake, buffering of potassium ions, coupling of gap junctions, and the release of cytokines and chemokines [5]. When these cytokines are released, A1 reactive astrocytes undergo a phenotypic shift leading to loss of their primary neuroprotective functions, such as the uptake of glutamate through the downregulation of EAAT2/GLT-1 transporters [35]. Consequently, interventions that upregulate glutamate transporters have shown promise in counteracting cytokine-mediated neuroinflammation and attenuating pain behavior [66,67]. Glutamate transport modulators (EAAT2/GLT-1 stimulators) can reduce the excessive synaptic glutamate accumulation and prevent the A1 astrogliosis development [68,69].

Pathological coupling of astrocytes via connexin-43 hemichannels has been considered as another mechanism involved in chronic pain [70]. The use of gap junction inhibitors targeting connexin-43 has been shown to modulate abnormal communication of astrocytes, reduce calcium waves found in glia, and consequently modulate excessive excitability of neurons [70,71]. Additional astrocyte-focused therapeutic strategies include CXCL1/CXCR2 inhibitors, which suppress chemokine-driven excitability and mitigate astrocyte-mediated sensitization [70,71].

### 6.4. Satellite Glial Cell (SGC)-Targeted Therapies

Satellite glial cells (SGCs) play a critical role in the amplification of peripheral sensitization [14,32]. Antagonists of P2Y2 and P2X7 purinergic receptors display profound anti-allodynic effects through the suppression of SGC activation and the abolition of pain behaviors in preclinical models of trigeminal and inflammatory pain [58,72]. The P2Y2 antagonist AR-C118925 and the P2X7 antagonist A740003 demonstrated the ability to diminish SGC sensitivity and alleviate pain behaviors in preclinical models in the rat [58,72,73].

Gap junction blockers, such as carbenoxolone, further inhibit pathological SGC-to-SGC and SGC-to-neuron communication, preventing the abnormal transmission of pain signals [32,38,39]. The administration of carbenoxolone at the level of the DRG inhibits the acute phase of nociception and the second phase of the formalin test, supporting its role in dampening sustained pain responses [73].

Antagonists of lysophosphatidic acid (LPA) receptors also suppress activation of SGCs and relieve the acute nociceptive response through the inhibition of LPA signals in the dorsal root ganglia [74]. Modulation of SGC activity via activation of the Gq-GPCR pathway and restoration of metalloproteinase signaling has been demonstrated to be analgesic in instances of inflammatory and chemotherapy-induced pain [75,76].

### 6.5. Multi-Targeted Therapies

As the field of therapies directed at glial cells continues to evolve, several multi-targeted therapies have emerged as well that aim to modulate a wider scope of neuroimmune pathways; rather than focusing on a singular glial subtype. For instance, gene therapy approaches, through the enhancement of anti-inflammatory cytokines IL-10, can have a profound analgesic effect in preclinical and early clinical research [77,78]. The introduction of the IL-10 gene through viral and non-viral vectors achieves a sustained analgesic effect through the modulation of the neuroimmune axis [78]. Gene therapy research aimed at peripheral nociceptors and genetic pain factors has been rapidly progressing as well, although it remains largely preclinical [79]. There have also been advancements in biomarker developments, with promise from combined multimodal methodologies, such as imaging, molecular, sensory, and neurophysiological modalities [80]. Expression of gene biomarkers in the blood and advanced neuroimaging continues to be investigated for use as standard in clinical settings [80,81]. In addition to biologic and gene-based approaches, neuromodulatory pharmacotherapies have emerged as a field of interest. In particular, esketamine has been highlighted as a potential strategy to modulate neuroinflammatory and glial mechanisms relevant to chronic pain states, including inhibition of microglial activation and inflammatory signaling beyond NMDA receptor antagonism. These effects support the broader concept that targeting glia-associated signaling may complement conventional analgesic pathways [82].

Mesenchymal stem cells (MSCs) and their derived exosomes further illustrate the potential of multi-pathway interventions. MSCs and their exosomes can confer analgesic, anti-inflammatory, and neuroregenerative effects in various models of neuropathic pain, osteoarthritis, and spinal cord injury [83,84]. The exosomes can facilitate axonal regrowth as well as directly modulate the excitability and neuroinflammation of sensory neurons [83,84,85]. Collectively, these therapeutic advances demonstrate great promise in the field of glial-focused therapies and will guide future development of clinically viable interventions.

## 7. Translational Gaps and Future Directions

There has been substantial progress in understanding contributions of glial cells to chronic pain; however, several knowledge gaps are yet to be addressed. Future directions should prioritize defining the temporal progression and regional specificity of glial activation to clarify how neuroinflammatory responses shift from adaptive injury signaling to persistent, pain-maintaining states. High-resolution approaches such as single-cell and spatial profiling, alongside emerging in vivo imaging tools, may help identify distinct glial phenotypes and early biomarkers of maladaptive transitions. Recognizing sex-specific and patient-specific differences in immune–glial signaling will also be critical for moving toward stratified or personalized pain management.

Therapeutically, there is growing interest in restoring neuroprotective glial functions rather than broadly suppressing inflammation. Emerging strategies target metabolic resilience, purinergic and TLR pathways, and intercellular communication via exosomes. Among these, A1 reactive astrocyte conversion inhibitors are particularly promising: by blocking IL-1α, TNF-α, and C1q, they preserve astrocytic neuroprotection and maintain EAAT2 expression. Although primarily studied in neurodegeneration, mechanistic overlap with chronic pain suggests they could serve as future disease-modifying analgesics. To our knowledge, no direct studies have tested A1-conversion inhibitors in chronic pain, and despite promising preclinical data, human trials are currently lacking, representing a major barrier to clinical translation that needs to be addressed.

## 8. Conclusions

Chronic pain is due to glia-driven neuroinflammatory dysregulation in microglia, astrocytes, and satellite glial cells. Loss of their neuroprotective functions combined with sustained inflammatory signaling leads to persistent sensitivity of peripheral and central pain pathways, resulting in chronic pain. Disruption of individual glial cell types contributes to a distinct role in chronic pain pathways, with microglia initiating neuroinflammation and central sensitization, astrocytes amplifying chronic pain through impaired glutamate and potassium homeostasis and cytokine release, and satellite glial cells driving peripheral sensitization through enhanced neuron–glia coupling and inflammatory mediator production. Understanding the role of glial pathway dysregulation in chronic pain and the transition of glial cells from neuroprotective to harmful phenotypes provides a target for non-opioid, glial-targeted therapies that aim to restore neuroprotective function. Current limitations include a lack of unified frameworks that address how different glial cell types simultaneously influence chronic pain as well as reliable human biomarkers of glial activation that signify neuroinflammation. However, development of these mechanisms offers potential for future therapies that achieve durable relief in addition to restoring glial homeostasis, preventing neuroinflammatory progression, and ultimately transforming care in patients with chronic pain.

## Figures and Tables

**Table 1:** Key glial mediators, mechanisms, and clinical relevance in chronic pain.

Glial Cell Type	Major Mediators	Mechanistic Effect	Neuroprotection Function Lost	Clinical Relevance
Microglia	IL-1β, TNF-α, BDNF	Central sensitization	Impaired debris clearance; loss of anti-inflammatory M2 phenotype; oxidative stress	Neuropathic pain
Astrocytes	CXCL1, CCL2 (MCP-1), IL-6, EAAT2 ↓, ATP	Persistent excitability	Loss of metabolic support; impaired glutamate uptake; impaired potassium buffering	Chronic back pain
Satellite Glial cells	IL-6, Cx43	DRG cross-excitation	Loss of ion buffering; reduced ganglionic insulation; impaired inflammatory control	Orofacial pain

**Table 2:** Novel therapeutic targets.

Target	Mechanism	Glial Cell Type	Strategy
TLR4	DAMP-driven	Microglia	TLR-4 inhibitors
P2X4/P2X7	ATP-dependent purinergic activation	Microglia	Antagonists
NF-κB	Pro-inflammatory transcription	Microglia/astrocytes	NF-κB inhibitors
CSF1R	Proliferation and activation	Microglia	CSF1R inhibitors
Minocycline	Broad glial suppression	Microglia	Repurposed drug
NLRP3	IL-1β maturation	Microglia/astrocytes	Inflammasome inhibitors
CXCL1/CXCR2	Chemokine-driven excitability	Astrocytes	CXCL1/CXCR2 inhibitors
EAAT2	Impaired glutamate uptake	Astrocytes	EAAT2 upregulators
Microglial and astrocytic inflammatory signaling pathways	Modulates glial inflammatory signaling	Microglia/astrocytes	MSC-derived exosomes

ATP: adenosine triphosphate; TLR4: toll-like receptor 4; NLRP3: nucleotide-binding domain leucine-rich repeat-containing protein 3.

## Data Availability

No new data were created or analyzed in this study.

## Abbreviations

ATP	Adenosine Triphosphate
BDNF	Brain-Derived Neurotrophic Factor
C1q	Complement Component 1q
CCL2	C-C Motif Chemokine Ligand 2
CX3CL1	Fractalkine (C-X3-C Motif Chemokine Ligand 1)
CX3CR1	Fractalkine Receptor
CXCL1	C-X-C Motif Chemokine Ligand 1
CNS	Central Nervous System
CSF1R	Colony-Stimulating Factor 1 Receptor
Cx43	Connexin-43
DAMPs	Danger-Associated Molecular Patterns
DRG	Dorsal Root Ganglion/Dorsal Root Ganglia
EAAT2	Excitatory Amino Acid Transporter 2
ERK	Extracellular Signal-Regulated Kinase
GFAP	Glial Fibrillary Acidic Protein
GLT-1	Glutamate Transporter 1
Gq-GPCR	Gq protein-coupled receptor
HMGB1	High Mobility Group Box 1
IL-1α	Interleukin-1 alpha
IL-1β	Interleukin-1 beta
IL-4	Interleukin-4
IL-6	Interleukin-6
IL-10	Interleukin-10
JAK	Janus Kinase
KCC2	Potassium-Chloride Cotransporter 2
Kir4.1	Inward-Rectifying Potassium Channel 4.1
LPA	Lysophosphatidic Acid
MAPK	Mitogen-Activated Protein Kinase
M1/M2 Phenotypes	Classically activated/alternatively activated microglia
MCP-1	Monocyte Chemoattractant Protein-1
MSCs	Mesenchymal Stem Cells
NF-κB	Nuclear Factor kappa-light-chain-enhancer of activated B cells
NLRP3	NOD-like Receptor Family Pyrin Domain Containing 3
NMDA receptor	N-methyl-D-aspartate Receptor
P2X4	Purinergic Receptor P2X4
P2X7	Purinergic Receptor P2X7
P2Y	Purinergic Receptor P2Y
p38 MAPK	p38 Mitogen-Activated Protein Kinase
PNS	Peripheral Nervous System
PPAR-γ	Peroxisome Proliferator-Activated Receptor gamma
PRRs	Pattern Recognition Receptors
ROS	Reactive Oxygen Species
SGCs	Satellite Glial Cells
S100β	S100 Calcium-Binding Protein Beta
STAT3	Signal Transducer and Activator of Transcription 3
TLR4	Toll-Like Receptor 4
TNF-α	Tumor Necrosis Factor alpha
TrkB	Tropomyosin Receptor Kinase B
TSPO-PET	Translocator Protein Positron Emission Tomography
